# Structural Entanglement from Interaction-Induced Fixed Points

**DOI:** 10.3390/e28070757

**Published:** 2026-07-02

**Authors:** Yukio-Pegio Gunji, Andrei Khrennikov

**Affiliations:** 1 Intermedia Art and Science, School of Fundamental Science and Technology, Waseda University, Ohkubo 3-4-1, Shinjuku-ku, Tokyo 169-8555, Japan; 2 Department of Mathematics, Linnaeus University, Universitetsplatsen-1, 352 52 Växjö, Sweden; andrei.khrennikov@lnu.se

**Keywords:** quantum logic, entanglement, tensor product, lattice theory, rough set

## Abstract

We introduce a lattice-theoretic framework for composite information systems in which tensor-like composition and entanglement are defined without presupposing Hilbert spaces or quantum states. Starting from approximation operators induced by indiscernibility relations, we construct composite systems via interaction-dependent closure operators and characterize their fixed-point lattices. Entanglement is defined structurally as the impossibility of generating a fixed point of the composite system from local fixed points alone. This notion does not rely on non-distributive logic a priori and remains meaningful even when local lattices are Boolean. Non-distributive and orthomodular structures arise only under additional conditions and are treated as emergent properties rather than assumptions. The proposed framework generalizes the concept of entanglement as a property of composition and interaction, providing a unified information-theoretic perspective on non-separability beyond standard quantum-mechanical formalisms. By mapping quantum states to correlation patterns via row-set tensor products, we demonstrate that standard quantum entanglement can be understood as a stabilized structural constraint. In this context, maximally entangled states, such as Bell states, correspond to diagonal constraint sets that are non-generable from local components, confirming that the structural core of entanglement exists independently of linear or probabilistic interpretations. Beyond quantum mechanics, the framework admits a natural interpretation in terms of relational databases, where entanglement corresponds to irreducible global relations stabilized by interaction-induced fixed points.

## 1. Introduction

Entanglement plays a central role in quantum information theory, characterizing forms of non-separability that cannot be reduced to local descriptions [1,2,3]. While standard formulations rely on tensor products of Hilbert spaces [4], there has been increasing interest in understanding entanglement as a more general structural property of composite systems, independent of specific physical realizations [5,6,7]. Such perspectives are relevant not only to quantum foundations but also to generalized information theory, contextuality, and abstract models of computation [8,9,10,11].

In this work, we propose a lattice-theoretic approach to entanglement based on fixed points of approximation operators induced by indiscernibility relations [12,13,14,15,16]. Rather than assuming non-distributive logic or quantum states from the outset [17,18,19], we focus on how interaction-dependent composition generates fixed points that cannot be constructed from local components alone [10,20]. This allows us to define tensor-like composition and entanglement at the level of information structure [11,20,21]. Non-distributive or orthomodular lattices arise only as special cases under additional constraints and are not presupposed [18,19].

To bridge the gap between abstract lattice theory and physical reality, this paper connects the concept of “fixed-point lattices” to the observable correlations found in bipartite quantum systems [7]. Standard quantum entanglement is typically defined by the non-factorizability of state vectors [1,4]; however, by utilizing row-sets to represent orthonormal bases, we can represent these states as combinatorial supports within a product universe [11,21]. This allows us to identify a “structural entanglement” that extracts and stabilizes the core correlation patterns of a state while abstracting away complex amplitudes and phases [9,10,22].

Through this lens, we show that a quantum state is structurally entangled if its associated support, after being processed by interaction-dependent approximation operators, cannot be reconstructed from its local embeddings [10]. This approach reveals that the non-separability of systems like the Bell state is fundamentally a result of diagonal constraint sets that defy local generation, providing a robust, information-theoretic foundation for non-locality [7,23].

Finally, we show that the proposed notion of structural entanglement admits a natural interpretation in terms of relational databases, where global relations stabilized by interaction-induced approximation operators cannot be generated from local projections alone. This perspective clarifies the relation between entanglement, contextuality, and irreducible information structures in composite systems.

The primary motivation of this work is to identify which aspects of entanglement are genuinely tied to Hilbert-space quantum mechanics and which aspects reflect a more general structural property of composite systems.

Standard formulations define entanglement through tensor-product decompositions of Hilbert spaces [1,4]. While extremely successful, such formulations make it difficult to distinguish features that are fundamentally quantum from those that arise more generally from interaction-induced constraints and non-separability.

The present work therefore investigates whether entanglement can be characterized at the level of information structure itself. By focusing on fixed points generated by interaction-dependent approximation operators, we obtain a notion of entanglement that remains meaningful even when local lattices are Boolean and no Hilbert-space representation is assumed.

### Relation to Existing Approaches

Several attempts have been made to generalize the notion of entanglement beyond its standard Hilbert-space formulation.

Generalized entanglement [24,25] characterizes non-separability with respect to distinguished observable algebras rather than subsystem decompositions. Generalized probabilistic theories (GPTs) [5,6] investigate entanglement and non-locality within operational frameworks that extend quantum theory. In categorical quantum mechanics [11,21], compositional structure is studied through monoidal categories and diagrammatic representations. Furthermore, contextuality and non-locality have been formulated in relational and sheaf-theoretic terms [10].

The present work is complementary to these approaches. Rather than starting from observable algebras, operational probabilistic models, categorical compositions, or presheaf structures, we focus on interaction-induced approximation operators and their fixed points. Entanglement is defined as the failure of global fixed points to be generated from local embeddings. Consequently, the framework applies even when local systems are represented by Boolean lattices and does not require Hilbert spaces, probability measures, or tensor-product structures as primitive assumptions.

## 2. Results

Our construction is based on rough-set approximation operators introduced by Pawlak [12,26]. In rough-set theory, a binary relation on a universe induces lower and upper approximation operators, which describe the sets that are certainly or possibly compatible with a given description. These operators provide a natural way to represent information structures under limited discernibility.

The interaction between lower and upper approximations generates a composite operator whose fixed points represent stable information states. Such fixed points have been extensively studied in rough-set theory and are known to form complete lattices under suitable conditions [27,28]. In the present work, we use these fixed-point lattices as the basic logical structures associated with individual systems. Composite systems are then constructed by introducing interaction-dependent relations between local systems and studying the fixed points that emerge from the resulting approximation operators.

From this perspective, entanglement is not introduced as a primitive notion. Instead, it emerges when the fixed-point lattice of the composite system contains elements that cannot be generated from fixed points of the local systems alone. We begin by reviewing the construction of fixed-point lattices for a single system.

### 2.1. Single System

The relations *R* and *K* represent indiscernibility structures in the sense of rough-set theory [12,13,14]. The operator $R_{*}$ corresponds to a lower approximation (certainty), whereas $K^{*}$ corresponds to an upper approximation (possibility). Their interaction generates stable information states represented by fixed points of the composite operator $T=R_{*}\circ K^{*}$.

**Definition** **1** (Approximation operator and fixed-point lattice)**.**

*Let U be a finite set. Let R and K be two binary relations on U, including the lower and upper approximation operators, $R_{*}:P\left(A\right)\to P\left(A\right)$ and $K^{*}:P\left(A\right)\to P\left(A\right)$ such that for any X⊆U*

(1) $$R_{*}\left(X\right)=\left\{x\in U|{\left[x\right]}_{R}\subseteq X\right\}$$

(2) $$K^{*}\left(X\right)=\left\{x\in U|{\left[x\right]}_{K}\cap X\neq ⌀\right\}.$$

**Proposition** **1.** *Define the composite approximation operator*

(3) $$T:=R_{*}\circ K^{*}$$

*The set of fixed points*

(4) L:=Fix(T)={x∈U∣T(X)=X}

*forms a complete lattice under set inclusion.*

The fact that fixed points of approximation operators form complete lattices is well known in rough-set theory and lattice theory [13,14].

### 2.2. Tensor-like Structure in Composite System

**Definition** **2** (Interaction-induced equivalence relations)**.** *Let $U_{A}$ and $U_{B}$ be finite universes. Let $R_{A},K_{A}$ be equivalence relations on $U_{A}$, and $R_{B},K_{B}$ equivalence relations on $U_{B}$. Let*

(5) $$\Gamma_{R},\Gamma_{K}\subseteq \left(U_{A}\times U_{B}\right)\times \left(U_{A}\times U_{B}\right)$$

*be interaction constraint relations. The interaction-induced equivalence relations on the composite universe*

(6) $$U_{AB}:=U_{A}\times U_{B}$$

*are defined by*

(7) $$R_{AB}:=\mathrm{EqCl}\left(\left(R_{A}\times R_{B}\right)\cup \Gamma_{R}\right),$$

(8) $$K_{AB}:=\mathrm{EqCl}\left(\left(K_{A}\times K_{B}\right)\cup \Gamma_{K}\right),$$

*where EqCl(·) denotes the reflexive, symmetric, and transitive closure.*

**Remark** **1.** *The original formulation based on a constraint set $C\subseteq U_{A}\times U_{B}$ is recovered as a special case of the present framework.*

*In Definition 2, the interaction constraint induces the relation*

(9) $$\bar{C}=C\times C\subseteq \left(U_{A}\times U_{B}\right)\times \left(U_{A}\times U_{B}\right).$$

*If*

(10) $$\Gamma_{R}=\Gamma_{K}=\bar{C},$$

*then*

(11) $$R_{AB}=\mathrm{EqCl}\left(\left(R_{A}\times R_{B}\right)\cup \bar{C}\right)=\left(R_{A}\times R_{B}\right)\vee \bar{C},$$

*and*

(12) $$K_{AB}=\mathrm{EqCl}\left(\left(K_{A}\times K_{B}\right)\cup \bar{C}\right)=\left(K_{A}\times K_{B}\right)\vee \bar{C}.$$

*Therefore, the original construction is obtained as the special case $\Gamma_{R}=\Gamma_{K}=\bar{C}$. The present formulation strictly generalizes the original framework by allowing independent interaction relations $\Gamma_{R}$ and $\Gamma_{K}$.*

This additional freedom makes it possible to construct fixed-point lattices that cannot be obtained from a single constraint set, including the diamond lattice $M_{3}$ presented in Section 2.6.

**Definition** **3** (Approximation operators in the composite system)**.** *For any subset $Z\subseteq U_{AB}$, the lower and upper approximation operators induced by $R_{AB}$ and $K_{AB}$ are defined by*

(13) $$R_{AB*}\left(Z\right)=\left\{x\in U_{AB}|{\left[x\right]}_{R_{AB}}\subseteq Z\right\},$$

(14) $${K_{AB}}^{*}\left(Z\right)=\left\{x\in U_{AB}|{\left[x\right]}_{K_{AB}}\cap Z\neq ⌀\right\}.$$

*The composite approximation operator is*

(15) $$T_{AB}=R_{AB*}\circ {K_{AB}}^{*}.$$

**Proposition** **2.**
(16) $$L_{AB}:=\left\{Z\subseteq U_{AB}|T_{AB}\left(Z\right)=Z\right\}=\mathrm{Fix}\left(T_{AB}\right).$$

*forms a complete lattice.*

### 2.3. Separability and Entanglement

**Definition** **4** (local embedding)**.**

*Let $L_{AB}:=\mathrm{Fix}\left(T_{AB}\right)$ be the fixed-point lattice of a composite system consisting of local fixed-point lattice such that*

(17) $$L_{A}:=\mathrm{Fix}\left(T_{A}\right),\;\;\;\;\;\;L_{B}:=\mathrm{Fix}\left(T_{B}\right),$$

*Local fixed points $X\in L_{A},Y\in L_{B}$ are embedded by*

(18) $$\iota_{A}\left(X\right):=T_{AB}\left(X\times U_{B}\right),\;\iota_{B}\left(Y\right):=T_{AB}\left(U_{A}\times Y\right).$$

*The sublattice generated by local embeddings is denoted by*

(19) $$S_{AB}:=\langle \iota_{A}\left(L_{A}\right)\cup \iota_{B}\left(L_{B}\right)\rangle$$

**Definition** **5** (entanglement)**.**

*An element $z\in L_{AB}$ is* separable *if*

(20) $$z\in S_{AB}.$$

*Otherwise, z is said to be* entangled.

**Remark** **2.** *This definition does not presuppose non-distributivity or orthomodularity. Entanglement is characterized solely by non-generability from local fixed points. Entanglement is defined as “inseparability for subalgebras” [24].*

**Proposition** **3.** *Let $L_{AB}=\mathrm{Fix}\left(T_{AB}\right)$ be the fixed-point lattice of the composite system. Then*

(21) $$S_{AB}:=\langle \iota_{A}\left(L_{A}\right)\cup \iota_{B}\left(L_{B}\right)\rangle$$

*is a sublattice of $L_{AB}$.*

**Proof.**  By definition, $\iota_{A}\left(X\right)=T_{AB}\left(X\times U_{B}\right)$ and $\iota_{B}\left(Y\right)=T_{AB}\left(U_{A}\times Y\right)$ are fixed points of $T_{AB}$ for all $X\in L_{A}$, $Y\in L_{B}$. Hence, $\iota_{A}\left(L_{A}\right)\cup \iota_{B}\left(L_{B}\right)\subseteq L_{AB}$. As $L_{AB}$ is a lattice, it is closed under finite joins and meets. Therefore, the sublattice generated by $\iota_{A}\left(L_{A}\right)\cup \iota_{B}\left(L_{B}\right)$ is contained in $L_{AB}$ and is itself closed under ∨ and ∧. Thus, $S_{AB}$ is a sublattice of $L_{AB}$. □

**Proposition** **4** (Sufficient condition for lattice homomorphisms)**.** *Assume that the composite fixed-point lattice $L_{AB}=\mathrm{Fix}\left(T_{AB}\right)$ is equipped with the lattice operations ∨,∧ induced from $\left(P\left(U_{AB}\right),\cup ,\cap \right)$. If $T_{AB}$ preserves finite unions and intersections, i.e., for all $Z,W\subseteq U_{AB}$,*

(22) $$T_{AB}\left(Z\cup W\right)=T_{AB}\left(Z\right)\vee T_{AB}\left(W\right),$$

(23) $$T_{AB}\left(Z\cap W\right)=T_{AB}\left(Z\right)\wedge T_{AB}\left(W\right),$$

*then the maps*

(24) $$\iota_{A}:L_{A}\to L_{AB},\;\iota_{A}\left(X\right)=T_{AB}\left(X\times U_{B}\right),$$

(25) $$\iota_{B}:L_{B}\to L_{AB},\;\iota_{B}\left(Y\right)=T_{AB}\left(U_{A}\times Y\right)$$

*are lattice homomorphisms.*

**Proof.**  Let $X,X^{\prime }\in L_{A}$. As $\left(X\cup X^{\prime }\right)\times U_{B}=\left(X\times U_{B}\right)\cup \left(X^{\prime }\times U_{B}\right)$, we have

$$\iota_{A}\left(X\vee X^{\prime }\right)=T_{AB}\left(\left(X\cup X^{\prime }\right)\times U_{B}\right)=T_{AB}\left(\left(X\times U_{B}\right)\cup \left(X^{\prime }\times U_{B}\right)\right)=\iota_{A}\left(X\right)\vee \iota_{A}\left(X^{\prime }\right).$$

Similarly, $\left(X\cap X^{\prime }\right)\times U_{B}=\left(X\times U_{B}\right)\cap \left(X^{\prime }\times U_{B}\right)$ implies

$$\iota_{A}\left(X\wedge X^{\prime }\right)=T_{AB}\left(\left(X\cap X^{\prime }\right)\times U_{B}\right)=T_{AB}\left(\left(X\times U_{B}\right)\cap \left(X^{\prime }\times U_{B}\right)\right)=\iota_{A}\left(X\right)\wedge \iota_{A}\left(X^{\prime }\right).$$

The proof for $\iota_{B}$ is analogous. □

**Proposition** **5** (Sufficient condition for $S_{AB}=L_{AB}$)**.** *Suppose that for every $z\in L_{AB}$ there exists a finite family ${\left\{\left(X_{k},Y_{k}\right)\right\}}_{k=1}^{n}$ with $X_{k}\in L_{A}$ and $Y_{k}\in L_{B}$ such that*

(26) $$z=\overset{n}{\underset{k=1}{⋁}}\left(\iota_{A}\left(X_{k}\right)\wedge \iota_{B}\left(Y_{k}\right)\right).$$

Then $S_{AB}=L_{AB}$.

**Proof.**  By definition, $S_{AB}$ is the smallest sublattice of $L_{AB}$ containing $\iota_{A}\left(L_{A}\right)\cup \iota_{B}\left(L_{B}\right)$, hence it is closed under finite meets and joins. Therefore each element of the form $\iota_{A}\left(X_{k}\right)\wedge \iota_{B}\left(Y_{k}\right)$ belongs to $S_{AB}$, and so does their finite join. Thus, $z\in S_{AB}$ for all $z\in L_{AB}$, which yields $L_{AB}\subseteq S_{AB}$. As $S_{AB}\subseteq L_{AB}$ always, we conclude $S_{AB}=L_{AB}$. □

**Remark** **3.** *In general, $S_{AB}\neq L_{AB}$ when the interaction-dependent interior operator (see [29] in physical interpretation) $R_{AB*}$ collapses or stabilizes global patterns that cannot be expressed as finite joins of rectangular generators $\iota_{A}\left(X\right)\wedge \iota_{B}\left(Y\right)$. This is precisely the mechanism behind the structurally entangled fixed points in Example 2.*

### 2.4. Minimal Examples of Structural Entanglement

In this section we present explicit computations of fixed-point lattices and entanglement for minimal composite systems. Throughout, entanglement is defined as *non-generability from local fixed points*.

#### 2.4.1. Example 1: 2×2 System Without Entanglement

Let

(27) $$U_{A}=\left\{a_{1},a_{2}\right\},\;U_{B}=\left\{b_{1},b_{2}\right\}.$$

Assume Boolean local lattices

(28) $$L_{A}=P\left(U_{A}\right),\;L_{B}=P\left(U_{B}\right).$$

Define the interaction-induced closure

(29) $${K_{AB}}^{*}\left(Z\right):=Z\cup C,\;C:=\left\{\left(a_{1},b_{1}\right),\left(a_{2},b_{2}\right)\right\},$$

and take a trivial interior

(30) $$R_{AB*}\left(Z\right):=Z.$$

Thus,

(31) $$T_{AB}\left(Z\right)=Z\cup C.$$

The fixed-point lattice is

(32) $$L_{AB}=\left\{Z\subseteq U_{AB}|C\subseteq Z\right\},$$

which consists of four elements:

(33) $$C,\;C\cup \left\{\left(a_{1},b_{2}\right)\right\},\;C\cup \left\{\left(a_{2},b_{1}\right)\right\},\;U_{AB}.$$

For any i,j, one computes

(34) $$\iota_{A}\left(\left\{a_{i}\right\}\right)\wedge \iota_{B}\left(\left\{b_{j}\right\}\right)=C\cup \left\{\left(a_{i},b_{j}\right)\right\}.$$

Hence, every fixed point is generated from local embeddings, and therefore

(35) $$S_{AB}=L_{AB}.$$

**Proposition** **6.** *The 2×2 system in Example 1 contains no entangled fixed points.*

#### 2.4.2. Example 2: 3×3 System with Boolean Local Lattices

Let

(36) $$U_{A}=\left\{a_{1},a_{2},a_{3}\right\},\;U_{B}=\left\{b_{1},b_{2},b_{3}\right\}.$$

Define

(37) $$D_{0}:=\left\{\left(a_{1},b_{1}\right),\left(a_{2},b_{2}\right),\left(a_{3},b_{3}\right)\right\},$$

and partition the remaining elements as

(38) $$D_{1}:=\left\{\left(a_{1},b_{2}\right),\left(a_{2},b_{3}\right),\left(a_{3},b_{1}\right)\right\},\;D_{2}:=\left\{\left(a_{1},b_{3}\right),\left(a_{2},b_{1}\right),\left(a_{3},b_{2}\right)\right\}.$$

Define

(39) $${K_{AB}}^{*}\left(Z\right):=Z\cup D_{0},$$

and let $R_{AB}$ be the equivalence relation whose equivalence classes are $D_{0},D_{1},D_{2}$. The lower approximation is

(40) $$R_{AB*}\left(W\right):=⋃\left\{D_{k}|D_{k}\subseteq W,\;k=0,1,2\right\}.$$

Thus,

(41) $$T_{AB}\left(Z\right)=R_{AB*}\left(Z\cup D_{0}\right).$$

The fixed-point lattice is

(42) $$L_{AB}=\left\{D_{0},\;D_{0}\cup D_{1},\;D_{0}\cup D_{2},\;U_{AB}\right\},$$

which forms a four-element diamond lattice.

Local embeddings satisfy

(43) $$\iota_{A}\left(L_{A}\right)=\iota_{B}\left(L_{B}\right)=\left\{D_{0},\;U_{AB}\right\};$$

hence,

(44) $$S_{AB}=\left\{D_{0},\;U_{AB}\right\}.$$

**Theorem** **1.** *The elements $D_{0}\cup D_{1}$ and $D_{0}\cup D_{2}$ are entangled fixed points in the 3×3 composite system, despite Boolean local lattices.*

These examples demonstrate that structural entanglement does not depend on non-distributive local logic, is absent in minimal systems with interaction-induced closure alone, and emerges when interaction constraints affect the interior approximation.

### 2.5. Explicit Construction of Structural Entanglement from Two Copies of $G_{12}$

We construct an explicit composite system from two local systems whose fixed-point lattices are isomorphic to the twelve-element Greechie lattice $G_{12}$. The purpose of this example is to show that even when the local systems and the composite system have the same lattice type, the composite fixed points need not be generated from local embeddings.

Let the local universes be

(45) $$U_{A}=\left\{a_{1},a_{2},a_{3},a_{4},a_{5}\right\},$$

and

(46) $$U_{B}=\left\{b_{1},b_{2},b_{3},b_{4},b_{5}\right\}.$$

The local fixed-point lattices are assumed to be the twelve-element Greechie lattice,

(47) $$L_{A}=\mathrm{Fix}\left(T_{A}\right)≃G_{12},$$

and

(48) $$L_{B}=\mathrm{Fix}\left(T_{B}\right)≃G_{12}.$$

Explicitly, the local lattice on $U_{A}$ is

(49) $$\begin{array}{cc} L_{A}= & \{⌀,\left\{a_{1}\right\},\left\{a_{2}\right\},\left\{a_{3}\right\},\left\{a_{4}\right\},\left\{a_{5}\right\},\\ & \;\;\{a_{1},a_{3}\},\left\{a_{2},a_{3}\right\},\left\{a_{3},a_{4}\right\},\left\{a_{3},a_{5}\right\},\\ & \;\left\{a_{1},a_{2},a_{4},a_{5}\right\},U_{A}\}. \end{array}$$

Similarly, the local lattice on $U_{B}$ is

(50) $$\begin{array}{cc} L_{B}= & \{⌀,\left\{b_{1}\right\},\left\{b_{2}\right\},\left\{b_{3}\right\},\left\{b_{4}\right\},\left\{b_{5}\right\},\\ & \;\;\left\{b_{1},b_{3}\right\},\left\{b_{2},b_{3}\right\},\left\{b_{3},b_{4}\right\},\left\{b_{3},b_{5}\right\},\\ & \;\;\left\{b_{1},b_{2},b_{4},b_{5}\right\},U_{B}\}. \end{array}$$

The composite universe is

(51) $$U_{AB}=U_{A}\times U_{B}.$$

Thus,

(52) $$|U_{AB}|=25.$$

We now define five correlation classes in the composite universe. These classes will serve as the $R_{AB}$-equivalence classes.

(53) $$E_{1}=\left\{\left(a_{1},b_{1}\right),\left(a_{2},b_{2}\right),\left(a_{3},b_{3}\right),\left(a_{4},b_{4}\right),\left(a_{5},b_{5}\right)\right\}.$$

(54) $$E_{2}=\left\{\left(a_{1},b_{2}\right),\left(a_{2},b_{3}\right),\left(a_{3},b_{4}\right),\left(a_{4},b_{5}\right),\left(a_{5},b_{1}\right)\right\}.$$

(55) $$E_{3}=\left\{\left(a_{1},b_{3}\right),\left(a_{2},b_{4}\right),\left(a_{3},b_{5}\right),\left(a_{4},b_{1}\right),\left(a_{5},b_{2}\right)\right\}.$$

(56) $$E_{4}=\left\{\left(a_{1},b_{4}\right),\left(a_{2},b_{5}\right),\left(a_{3},b_{1}\right),\left(a_{4},b_{2}\right),\left(a_{5},b_{3}\right)\right\}.$$

(57) $$E_{5}=\left\{\left(a_{1},b_{5}\right),\left(a_{2},b_{1}\right),\left(a_{3},b_{2}\right),\left(a_{4},b_{3}\right),\left(a_{5},b_{4}\right)\right\}.$$

These five sets form a partition of the composite universe:

(58) $$U_{AB}=E_{1}\cup E_{2}\cup E_{3}\cup E_{4}\cup E_{5}.$$

We define the $R_{AB}$-equivalence classes by

(59) $$U_{AB}/R_{AB}=\left\{E_{1},E_{2},E_{3},E_{4},E_{5}\right\}.$$

Next, we define five $K_{AB}$-equivalence classes.

(60) $$F_{1}=\left\{\left(a_{1},b_{1}\right),\left(a_{4},b_{4}\right),\left(a_{1},b_{4}\right),\left(a_{4},b_{2}\right),\left(a_{1},b_{5}\right),\left(a_{4},b_{3}\right)\right\}.$$

(61) $$F_{2}=\left\{\left(a_{2},b_{2}\right),\left(a_{5},b_{5}\right),\left(a_{1},b_{2}\right),\left(a_{4},b_{5}\right),\left(a_{2},b_{1}\right),\left(a_{5},b_{4}\right)\right\}.$$

(62) $$F_{3}=\left\{\left(a_{2},b_{3}\right),\left(a_{5},b_{1}\right),\left(a_{2},b_{5}\right),\left(a_{5},b_{3}\right),\left(a_{3},b_{2}\right)\right\}.$$

(63) $$F_{4}=\left\{\left(a_{3},b_{3}\right),\left(a_{3},b_{4}\right),\left(a_{3},b_{1}\right)\right\}.$$

(64) $$F_{5}=\left\{\left(a_{1},b_{3}\right),\left(a_{2},b_{4}\right),\left(a_{3},b_{5}\right),\left(a_{4},b_{1}\right),\left(a_{5},b_{2}\right)\right\}.$$

These sets also form a partition of $U_{AB}$:

(65) $$U_{AB}=F_{1}\cup F_{2}\cup F_{3}\cup F_{4}\cup F_{5}.$$

Thus,

(66) $$U_{AB}/K_{AB}=\left\{F_{1},F_{2},F_{3},F_{4},F_{5}\right\}.$$

The interaction between the two approximation structures is encoded by the incidence relation between the $R_{AB}$-classes and the $K_{AB}$-classes. For each $E_{i}$, define

(67) $$N\left(E_{i}\right)=\left\{F_{j}|E_{i}\cap F_{j}\neq ⌀\right\}.$$

From the definitions above, we obtain

(68) $$N\left(E_{1}\right)=\left\{F_{1},F_{2},F_{4}\right\},$$

(69) $$N\left(E_{2}\right)=\left\{F_{2},F_{3},F_{4}\right\},$$

(70) $$N\left(E_{3}\right)=\left\{F_{5}\right\},$$

(71) $$N\left(E_{4}\right)=\left\{F_{1},F_{3},F_{4}\right\},$$

and

(72) $$N\left(E_{5}\right)=\left\{F_{1},F_{2},F_{3}\right\}.$$

For any union of $R_{AB}$-classes,

(73) $$X_{I}=\underset{i\in I}{⋃}E_{i},$$

where

(74) I⊆{1,2,3,4,5},

the upper approximation with respect to $K_{AB}$ is

(75) $${K_{AB}}^{*}\left(X_{I}\right)=⋃\left\{F_{j}|F_{j}\cap X_{I}\neq ⌀\right\}.$$

Using the notation $N(E_{i})$, this can be written as

(76) $${K_{AB}}^{*}\left(X_{I}\right)=\underset{j\in {⋃}_{i\in I}N\left(E_{i}\right)}{⋃}F_{j}.$$

The lower approximation with respect to $R_{AB}$ is

(77) $$R_{AB*}\left(Y\right)=⋃\left\{E_{k}|E_{k}\subseteq Y\right\}.$$

Therefore, the composite approximation operator is

(78) $$T_{AB}=R_{AB*}\circ {K_{AB}}^{*}.$$

For a union $X_{I}$ of $R_{AB}$-classes, we have

(79) $$T_{AB}\left(X_{I}\right)=⋃\left\{E_{k}|N\left(E_{k}\right)\subseteq \underset{i\in I}{⋃}N\left(E_{i}\right)\right\}.$$

We now compute $T_{AB}$ for all $2^{5}=32$ unions of $R_{AB}$-classes. The results are as follows.

(80) $$T_{AB}\left(⌀\right)=⌀.$$

(81) $$T_{AB}\left(E_{1}\right)=E_{1}.$$

(82) $$T_{AB}\left(E_{2}\right)=E_{2}.$$

(83) $$T_{AB}\left(E_{3}\right)=E_{3}.$$

(84) $$T_{AB}\left(E_{4}\right)=E_{4}.$$

(85) $$T_{AB}\left(E_{5}\right)=E_{5}.$$

(86) $$T_{AB}\left(E_{1}\cup E_{2}\right)=E_{1}\cup E_{2}\cup E_{4}\cup E_{5}.$$

(87) $$T_{AB}\left(E_{1}\cup E_{3}\right)=E_{1}\cup E_{3}.$$

(88) $$T_{AB}\left(E_{1}\cup E_{4}\right)=E_{1}\cup E_{2}\cup E_{4}\cup E_{5}.$$

(89) $$T_{AB}\left(E_{1}\cup E_{5}\right)=E_{1}\cup E_{2}\cup E_{4}\cup E_{5}.$$

(90) $$T_{AB}\left(E_{2}\cup E_{3}\right)=E_{2}\cup E_{3}.$$

(91) $$T_{AB}\left(E_{2}\cup E_{4}\right)=E_{1}\cup E_{2}\cup E_{4}\cup E_{5}.$$

(92) $$T_{AB}\left(E_{2}\cup E_{5}\right)=E_{1}\cup E_{2}\cup E_{4}\cup E_{5}.$$

(93) $$T_{AB}\left(E_{3}\cup E_{4}\right)=E_{3}\cup E_{4}.$$

(94) $$T_{AB}\left(E_{3}\cup E_{5}\right)=E_{3}\cup E_{5}.$$

(95) $$T_{AB}\left(E_{4}\cup E_{5}\right)=E_{1}\cup E_{2}\cup E_{4}\cup E_{5}.$$

(96) $$T_{AB}\left(E_{1}\cup E_{2}\cup E_{3}\right)=E_{1}\cup E_{2}\cup E_{3}\cup E_{4}\cup E_{5}.$$

(97) $$T_{AB}\left(E_{1}\cup E_{2}\cup E_{4}\right)=E_{1}\cup E_{2}\cup E_{4}\cup E_{5}.$$

(98) $$T_{AB}\left(E_{1}\cup E_{2}\cup E_{5}\right)=E_{1}\cup E_{2}\cup E_{4}\cup E_{5}.$$

(99) $$T_{AB}\left(E_{1}\cup E_{3}\cup E_{4}\right)=E_{1}\cup E_{2}\cup E_{3}\cup E_{4}\cup E_{5}.$$

(100) $$T_{AB}\left(E_{1}\cup E_{3}\cup E_{5}\right)=E_{1}\cup E_{2}\cup E_{3}\cup E_{4}\cup E_{5}.$$

(101) $$T_{AB}\left(E_{1}\cup E_{4}\cup E_{5}\right)=E_{1}\cup E_{2}\cup E_{4}\cup E_{5}.$$

(102) $$T_{AB}\left(E_{2}\cup E_{3}\cup E_{4}\right)=E_{1}\cup E_{2}\cup E_{3}\cup E_{4}\cup E_{5}.$$

(103) $$T_{AB}\left(E_{2}\cup E_{3}\cup E_{5}\right)=E_{1}\cup E_{2}\cup E_{3}\cup E_{4}\cup E_{5}.$$

(104) $$T_{AB}\left(E_{2}\cup E_{4}\cup E_{5}\right)=E_{1}\cup E_{2}\cup E_{4}\cup E_{5}.$$

(105) $$T_{AB}\left(E_{3}\cup E_{4}\cup E_{5}\right)=E_{1}\cup E_{2}\cup E_{3}\cup E_{4}\cup E_{5}.$$

(106) $$T_{AB}\left(E_{1}\cup E_{2}\cup E_{3}\cup E_{4}\right)=E_{1}\cup E_{2}\cup E_{3}\cup E_{4}\cup E_{5}.$$

(107) $$T_{AB}\left(E_{1}\cup E_{2}\cup E_{3}\cup E_{5}\right)=E_{1}\cup E_{2}\cup E_{3}\cup E_{4}\cup E_{5}.$$

(108) $$T_{AB}\left(E_{1}\cup E_{2}\cup E_{4}\cup E_{5}\right)=E_{1}\cup E_{2}\cup E_{4}\cup E_{5}.$$

(109) $$T_{AB}\left(E_{1}\cup E_{3}\cup E_{4}\cup E_{5}\right)=E_{1}\cup E_{2}\cup E_{3}\cup E_{4}\cup E_{5}.$$

(110) $$T_{AB}\left(E_{2}\cup E_{3}\cup E_{4}\cup E_{5}\right)=E_{1}\cup E_{2}\cup E_{3}\cup E_{4}\cup E_{5}.$$

(111) $$T_{AB}\left(E_{1}\cup E_{2}\cup E_{3}\cup E_{4}\cup E_{5}\right)=E_{1}\cup E_{2}\cup E_{3}\cup E_{4}\cup E_{5}.$$

Therefore, the fixed points of $T_{AB}$ are exactly

(112) $$\begin{array}{cc} L_{AB}= & \{⌀,E_{1},E_{2},E_{3},E_{4},E_{5},\\ & \;\;E_{1}\cup E_{3},E_{2}\cup E_{3},E_{3}\cup E_{4},E_{3}\cup E_{5},\\ & \;\;E_{1}\cup E_{2}\cup E_{4}\cup E_{5},U_{AB}\}. \end{array}$$

Thus,

(113) $$|L_{AB}|=12.$$

Moreover, this fixed-point lattice is isomorphic to the Greechie lattice $G_{12}$:

(114) $$L_{AB}≃G_{12}.$$

We now compute the locally generated sublattice. The local embeddings are defined as

(115) $$\iota_{A}\left(X\right)=T_{AB}\left(X\times U_{B}\right),$$

and

(116) $$\iota_{B}\left(Y\right)=T_{AB}\left(U_{A}\times Y\right).$$

Each $E_{i}$ projects onto the whole of $U_{A}$:

(117) $$\pi_{A}\left(E_{i}\right)=U_{A}.$$

Similarly, each $E_{i}$ projects onto the whole of $U_{B}$:

(118) $$\pi_{B}\left(E_{i}\right)=U_{B}.$$

Therefore, for every non-empty subset $X\subseteq U_{A}$,

(119) $$E_{i}\cap \left(X\times U_{B}\right)\neq ⌀$$

for every

(120) i=1,2,3,4,5.

Hence, for every non-empty $X\in L_{A}$,

(121) $$T_{AB}\left(X\times U_{B}\right)=U_{AB}.$$

On the other hand,

(122) $$T_{AB}\left(⌀\times U_{B}\right)=T_{AB}\left(⌀\right)=⌀.$$

Therefore,

(123) $$\iota_{A}\left(L_{A}\right)=\left\{⌀,U_{AB}\right\}.$$

The same argument gives

(124) $$\iota_{B}\left(L_{B}\right)=\left\{⌀,U_{AB}\right\}.$$

Thus, the sublattice generated by local embeddings is

(125) $$S_{AB}=\langle \iota_{A}\left(L_{A}\right)\cup \iota_{B}\left(L_{B}\right)\rangle .$$

Substituting the computed embeddings, we obtain

(126) $$S_{AB}=\langle \{⌀,U_{AB}\}\rangle .$$

Hence,

(127) $$S_{AB}=\left\{⌀,U_{AB}\right\}.$$

Consequently,

(128) $$|S_{AB}|=2.$$

The structurally entangled fixed points are

(129) $$L_{AB}\setminus S_{AB}.$$

Therefore,

(130) $$\begin{array}{cc} L_{AB}\setminus S_{AB}= & \{E_{1},E_{2},E_{3},E_{4},E_{5},\\ & \;\;E_{1}\cup E_{3},E_{2}\cup E_{3},E_{3}\cup E_{4},E_{3}\cup E_{5},\\ & \;\;E_{1}\cup E_{2}\cup E_{4}\cup E_{5}\}. \end{array}$$

Thus,

(131) $$|L_{AB}\setminus S_{AB}|=10.$$

This example shows that even though

(132) $$L_{A}≃G_{12},$$

(133) $$L_{B}≃G_{12},$$

and

(134) $$L_{AB}≃G_{12},$$

the locally generated sublattice collapses to the two-element lattice

(135) $$S_{AB}=\left\{⌀,U_{AB}\right\}.$$

Thus, all non-trivial elements of the composite $G_{12}$ lattice are structurally entangled. This demonstrates that structural entanglement is not a consequence of the size or non-distributivity of the local lattices alone, but of the interaction-induced mismatch between the local embeddings and the global fixed-point structure.

**Figure 1:**
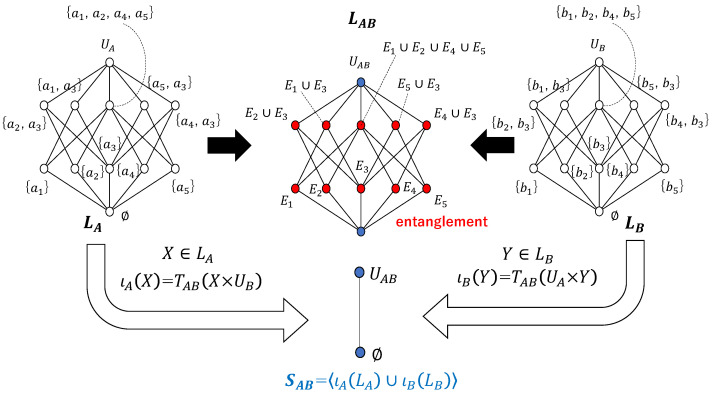
Structural entanglement in the composite system of two $G_{12}$ lattices. The local fixed-point lattices $L_{A}$ and $L_{B}$ are both isomorphic to the twelve-element Greechie lattice $G_{12}$. The composite approximation operator $T_{AB}=R_{AB*}\circ {K_{AB}}^{*}$ induces a global fixed-point lattice $L_{AB}$ that is again isomorphic to $G_{12}$. Local fixed points are embedded into the composite system through $\iota_{A}\left(X\right)=T_{AB}\left(X\times U_{B}\right)$ and $\iota_{B}\left(Y\right)=T_{AB}\left(U_{A}\times Y\right)$. Because every correlation class projects onto both local universes, the locally generated sublattice collapses to $S_{AB}=\langle \iota_{A}\left(L_{A}\right)\cup \iota_{B}\left(L_{B}\right)\rangle =\left\{⌀,U_{AB}\right\}$. Consequently, all non-trivial elements of $L_{AB}$ (shown in red) belong to $L_{AB}\setminus S_{AB}$ and are structurally entangled. In this example, $|L_{AB}|=12$, $|S_{AB}|=2$, and $|L_{AB}\setminus S_{AB}|=10$.

This example shows that the emergence of entanglement is not a consequence of Hilbert-space superposition, but, rather, of interaction-induced fixed points that cannot be generated from local contextual structures alone, as shown in Figure 1.

- Local Logics ($L_{A},L_{B}$): Each local system is described by the twelve-element Greechie lattice $G_{12}$. The lattice contains five atoms and twelve elements in total, and is non-distributive.
- Composite Universe ($U_{AB}$): The composite universe is defined as
  $$U_{AB}=U_{A}\times U_{B},$$
  with
  $$|U_{A}\left|=\right|U_{B}\left|=5,\;\right|U_{AB}|=25.$$
- Interaction Structure: The interaction is not specified by a Hardy-type condition but by a non-trivial incidence relation between the $R_{AB}$-equivalence classes $\left\{E_{1},\ldots ,E_{5}\right\}$ and the $K_{AB}$-equivalence classes $\left\{F_{1},\ldots ,F_{5}\right\}$. This interaction defines the composite approximation operator
  $$T_{AB}=R_{AB*}\circ {K_{AB}}^{*}.$$
- Composite Logic: The fixed-point lattice
  $$L_{AB}=\mathrm{Fix}\left(T_{AB}\right)$$
  contains twelve elements and is isomorphic to the same Greechie lattice $G_{12}$.
- Structural Entanglement: The local embeddings generate only
  $$S_{AB}=\left\{⌀,U_{AB}\right\}.$$
  Consequently,
  $$L_{AB}\setminus S_{AB}$$
  contains ten elements.
- Result: Although
  $$L_{A}≃L_{B}≃L_{AB}≃G_{12},$$
  the locally generated structure collapses to a two-element lattice. Hence, every non-trivial element of the composite Greechie lattice is structurally entangled. The entanglement arises entirely from the interaction-induced fixed-point structure and does not require wavefunctions, amplitudes, or Hilbert-space superposition.

### 2.6. Extension: Cognitive Modeling and the Structural Emergence of Psychological Non-Distributivity

The lattice-theoretic framework of interaction-induced fixed points and structural entanglement established in this work has profound implications beyond quantum foundations, offering a non-probabilistic approach to cognitive science and quantum-like modeling of human decision- aking [30,31,32]. Recent empirical research in cognitive psychology demonstrates that human reasoning systematically violates classical Boolean logic, manifesting severe question order effects (QOEs) [33] and violations of the response replicability effect (RRE) [34,35].

Conventionally, these psychological anomalies are modeled by postulating a mental Hilbert space where cognitive states undergo projective transformations or evolve via quantum instruments [35,36]. However, by applying the structural entanglement framework developed in Section 2.2, we show that these non-classical cognitive shifts can be explained strictly as a property of stable information states arising from overlapping, interactive contexts, bypassing the need for complex amplitudes or state vectors.

Consider a human agent processing two distinct conceptual evaluation schemas or question spaces, represented as local systems $\left(U_{A},R_{A},K_{A}\right)$ and $\left(U_{B},R_{B},K_{B}\right)$. Locally, the agent’s baseline understanding of each independent schema is classical and perfectly distributive; the local indiscernibility relations yield standard Boolean lattices $L_{A}=P\left(U_{A}\right)$ and $L_{B}=P\left(U_{B}\right)$ as in Section 2.4.2. In isolation, an agent’s response to an inquiry within domain *A* is modeled by a local fixed point $X\in L_{A}$, representing a stable, reproducible cognitive state where the composite local approximation operator satisfies $T_{A}\left(X\right)=X$.

The transition to quantum-like behavior occurs when these two schemas are brought into simultaneous consideration during sequential or complex decision-making tasks. This cognitive synthesis is governed by an interaction constraint set $C\subseteq U_{A}\times U_{B}$, which encodes the psychological context, framing biases, or semantic overlap between the domains. The composite cognitive approximation operator $T_{AB}=R_{AB*}\circ {K_{AB}}^{*}$, altered by these real-world constraints, characterizes the global fixed-point lattice $L_{AB}=\mathrm{Fix}\left(T_{AB}\right)$.

As established by Ozawa and Khrennikov [34], the mathematical non-distributivity of human logic is fundamentally equivalent to the incompatibility of logical variables—the impossibility of simultaneously assigning joint, classical truth values to separate concepts in a single mental state. In our framework, when cognitive constraints are highly restrictive, the global lattice $L_{AB}$ collapses into a non-distributive structure (such as the four-element diamond or a Peterson–Greechie $G_{12}$ architecture) (See Appendix A). Crucially, the local lattices remain Boolean, demonstrating that psychological non-distributivity is an emergent property triggered entirely by cross-contextual interaction.

This structural model provides a clean, information-theoretic mechanism for the trade-off between the response replicability effect (RRE) and the question order effect (QOE). The RRE states that if an agent answers question *A* and is immediately asked question *A* again, the answer is replicated with certainty, which corresponds to the system settling into a stable fixed point ($T_{A}\left(X\right)=X$). However, if an intermediate question *B* is introduced between the two instances of *A*, human responses frequently flip, violating the RRE.

Under the projective update conjecture analyzed by Ozawa and Khrennikov, satisfying the RRE under all states mathematically forces the underlying logic to be distributive, making it incompatible with QOE [34]. In our framework, the failure of the RRE is a direct byproduct of navigating an entangled lattice $L_{AB}$. When $L_{AB}$ contains structurally entangled fixed points $Z\in L_{AB}\setminus S_{AB}$ that cannot be generated as a join or meet of local components ($\iota_{A}\left(X\right)\wedge \iota_{B}\left(Y\right)$), evaluating question *A*, then shifting focus to evaluate context *B*, forces a structural stabilization step via $T_{AB}$. Because the global operator does not preserve simple rectangular factorization under tight constraints, the sequential evaluation induces a logical “leak” across the overlapping context boundaries of the lattice. The system is systematically guided away from the original local fixed point, driving a transition to a different global fixed point.

Ultimately, this structural perspective reconciles the no-go boundaries of cognitive projective measurements. It reveals that the quantum-like nature of human logic is not an inherent quirk of mental wave functions, but, rather, the natural mathematical consequence of stable relational fixed points emerging from tightly constrained, interactive contextual systems.

It is straightforward to construct the diamond lattice $M_{3}$ from a composite system of two Boolean algebras. Let the sets of atoms of the two Boolean algebras be $U_{A}=\left\{a_{1},a_{2},a_{3}\right\},$ and $U_{B}=\left\{b_{1},b_{2},b_{3}\right\},$ and define each Boolean algebra as the corresponding power set.

The composite system is constructed by collecting all fixed points of

(136) $$T_{AB}\left(X\right)=R_{AB*}\;\left({K_{AB}}^{*}\left(X\right)\right)$$

for all subsets of $U_{AB}=U_{A}\times U_{B}.$ As $L_{A}$ and $L_{B}$ are the power set of $U_{A}$ and $U_{B}$, respectively,

(137) $$R_{A}=K_{A}=\Delta_{A},\;R_{B}=K_{B}=\Delta_{B},$$

where $\Delta_{A}$ and $\Delta_{B}$ denote the identity relations. Define the interaction relations

(138) $$\Gamma_{R}=\left\{\begin{array}{c} \left(\left(a_{1},b_{1}\right),\left(a_{2},b_{2}\right)\right),\left(\left(a_{2},b_{2}\right),\left(a_{3},b_{3}\right)\right),\\ \left(\left(a_{1},b_{2}\right),\left(a_{2},b_{3}\right)\right),\left(\left(a_{2},b_{3}\right),\left(a_{3},b_{1}\right)\right),\\ \left(\left(a_{1},b_{3}\right),\left(a_{2},b_{1}\right)\right),\left(\left(a_{2},b_{1}\right),\left(a_{3},b_{2}\right)\right) \end{array}\right\}.$$

and

(139) $$\Gamma_{K}=\left\{\begin{array}{c} \left(\left(a_{1},b_{3}\right),\left(a_{2},b_{1}\right)\right),\left(\left(a_{2},b_{1}\right),\left(a_{3},b_{1}\right)\right),\\ \left(\left(a_{1},b_{1}\right),\left(a_{2},b_{2}\right)\right),\left(\left(a_{2},b_{2}\right),\left(a_{3},b_{2}\right)\right),\\ \left(\left(a_{1},b_{2}\right),\left(a_{2},b_{3}\right)\right),\left(\left(a_{2},b_{3}\right),\left(a_{3},b_{3}\right)\right) \end{array}\right\}.$$

The interaction-induced equivalence relations are

(140) $$R_{AB}=\mathrm{EqCl}\left(\left(R_{A}\times R_{B}\right)\cup \Gamma_{R}\right),$$

(141) $$K_{AB}=\mathrm{EqCl}\left(\left(K_{A}\times K_{B}\right)\cup \Gamma_{K}\right).$$

As $R_{A}\times R_{B}=\Delta_{U_{AB}}$ and $K_{A}\times K_{B}=\Delta_{U_{AB}}$, the equivalence closure generates the following equivalence classes:

(142) $$U_{AB}/R_{AB}=\left\{D_{0},D_{1},D_{2}\right\},$$

where

(143) $$D_{0}=\left\{\left(a_{1},b_{1}\right),\left(a_{2},b_{2}\right),\left(a_{3},b_{3}\right)\right\},$$

(144) $$D_{1}=\left\{\left(a_{1},b_{2}\right),\left(a_{2},b_{3}\right),\left(a_{3},b_{1}\right)\right\},$$

(145) $$D_{2}=\left\{\left(a_{1},b_{3}\right),\left(a_{2},b_{1}\right),\left(a_{3},b_{2}\right)\right\},$$

and

(146) $$U_{AB}/K_{AB}=\left\{E_{0},E_{1},E_{2}\right\},$$

where

(147) $$E_{0}=\left\{\left(a_{1},b_{3}\right),\left(a_{2},b_{1}\right),\left(a_{3},b_{1}\right)\right\},$$

(148) $$E_{1}=\left\{\left(a_{1},b_{1}\right),\left(a_{2},b_{2}\right),\left(a_{3},b_{2}\right)\right\},$$

(149) $$E_{2}=\left\{\left(a_{1},b_{2}\right),\left(a_{2},b_{3}\right),\left(a_{3},b_{3}\right)\right\}.$$

The interaction relations therefore induce the incidence structure

(150) $$I_{AB}\subseteq \left(U_{AB}/R_{AB}\right)\times \left(U_{AB}/K_{AB}\right),$$

defined by

(151) $$D_{i}\;I_{AB}\;E_{j}\Leftrightarrow D_{i}\cap E_{j}\neq ⌀.$$

Thus, the incidence relation is not introduced independently but is induced by the interaction-generated equivalence classes of $R_{AB}$ and $K_{AB}$. Using this incidence structure,

(152) $${K_{AB}}^{*}\left(D_{0}\right)=E_{1}\cup E_{2},$$

and therefore

(153) $$T_{AB}\left(D_{0}\right)=R_{AB*}\;\left({K_{AB}}^{*}\left(D_{0}\right)\right)=R_{AB*}\left(E_{1}\cup E_{2}\right)=D_{0}.$$

Hence, $D_{0}$ is a fixed point. Similarly,

(154) $$T_{AB}\left(D_{1}\right)=D_{1},\;T_{AB}\left(D_{2}\right)=D_{2}.$$

On the other hand,

(155) $$T_{AB}\left(D_{0}\cup D_{1}\right)=U_{AB},$$

and similarly for any pair of distinct $D_{i}$ and $D_{j}$. Therefore, the fixed-point lattice is

(156) $$L_{AB}=\left\{⌀,D_{0},D_{1},D_{2},U_{AB}\right\},$$

which is isomorphic to the diamond lattice

(157) $$L_{AB}≅M_{3}.$$

**Figure 2:**
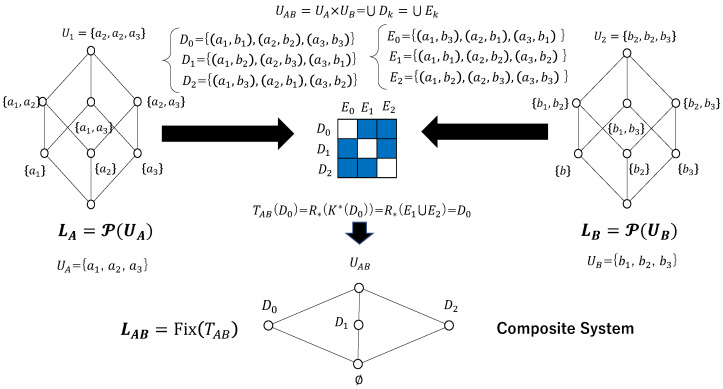
Emergence of the diamond lattice $M_{3}$ from interaction-induced structural gluing. Starting from two Boolean local systems $L_{A}=P\left(U_{A}\right)$ and $L_{B}=P\left(U_{B}\right)$, the interaction relations $\Gamma_{R}$ and $\Gamma_{K}$ generate distinct composite equivalence relations $R_{AB}$ and $K_{AB}$. Their equivalence classes $\left\{D_{i}\right\}$ and $\left\{E_{j}\right\}$ induce an incidence structure through non-empty intersections, represented by a blue square. The resulting approximation operator $T_{AB}=R_{AB*}\circ {K_{AB}}^{*}$ has a fixed-point lattice $L_{AB}$ consisting of $\left\{\emptyset ,D_{0},D_{1},D_{2},U_{AB}\right\}$, which is isomorphic to the diamond lattice $M_{3}$.

Figure 2 illustrates the construction process

(158) $$\Gamma_{R},\Gamma_{K}⟹R_{AB},K_{AB}⟹\left\{D_{i}\right\},\left\{E_{j}\right\}⟹I_{AB}⟹L_{AB}≅M_{3}.$$

**Proposition** **7.** *The fixed-point lattice $L_{AB}$ obtained from the above construction is isomorphic to the diamond lattice $M_{3}$.*

## 3. Discussion

From a physical perspective, structural entanglement can be understood as a stabilized correlation pattern that survives the interaction-induced approximation process. In contrast to conventional quantum theory, where entanglement is defined through non-factorizable state vectors, the present framework identifies entanglement directly at the level of admissible global structures. The resulting fixed points represent physically accessible correlation constraints rather than wavefunctions themselves. In this sense, structural entanglement describes the persistence of global organization that cannot be reconstructed from local information alone. In this section, we discuss how several concepts discussed in quantum theory can be interpreted using our proposed definition.

First, we discuss the relationship with row-set-based arguments and give examples of Bell-type correlation.

We define and realize entanglement using only finite orthogonal modular lattices, without using Hilbert spaces at all. However, when considering composite systems using Peterson–Greechie lattices, we can clarify that entanglement is not a superposition of states but, rather, a loop of contexts.

We also consider Hardy-type constraints. These constraints recognize a set of outcomes as “possible”, but define a set of alternative outcomes that are logically connected to it as forbidden events. While Hardy-type constraints are usually defined as probability conditions, in this chapter we present them as the complement of forbidden sets. We interpret Hardy-type constraints as cross-contextual forbidden relations imposed between local logics with non-distributive context structures, and as minimal conditions that necessarily generate global fixed points (structural entanglement) that cannot be recovered by the Cartesian product of local logic (also see Appendix B).

We will also discuss the new information theory that develops from this.

### 3.1. Relation to Standard Quantum Entanglement via Row Sets

For bipartite quantum systems, entangled states are characterized by non-factorizable correlation patterns between measurement outcomes. These patterns can be represented as subsets of outcome pairs (row–column relations). In particular, maximally entangled states correspond to diagonal-like constraint sets, which give rise to interaction-induced fixed points that are not generable from local row or column sets alone. In this sense, our definition captures the structural core of quantum entanglement at the level of observable correlations.

In this section we clarify how standard quantum entanglement can be related to our lattice-theoretic notion of structural entanglement by means of row sets and correlation patterns.

#### 3.1.1. Row Sets and Combinatorial Tensor Products

Let $H_{A}$ and $H_{B}$ be finite-dimensional Hilbert spaces. Let

(159) $$R=\left\{r_{1},\ldots ,r_{m}\right\}\subset H_{A},\;S=\left\{s_{1},\ldots ,s_{n}\right\}\subset H_{B}$$

be orthonormal bases. The row-set tensor product is defined as

(160) $$R⊗S:=\{\;r_{i}⊗s_{j}|1\leq i\leq m,\;1\leq j\leq n\;\}.$$

As sets, we identify

(161) R⊗S≅R×S.

Accordingly, we set

(162) $$U_{A}:=R,\;U_{B}:=S,\;U_{AB}:=U_{A}\times U_{B}.$$

#### 3.1.2. From Quantum States to Correlation Patterns

Let $|\psi \rangle \in H_{A}⊗H_{B}$ be a pure state, expanded as

(163) $$|\psi \rangle =\sum_{i=1}^{m}\sum_{j=1}^{n}\alpha_{ij}\;|r_{i}\rangle ⊗|s_{j}\rangle .$$

We define the support (or correlation pattern) of |ψ〉 by

(164) $$\mathrm{Supp}\left(\psi \right):=\{\left(r_{i},s_{j}\right)\in U_{AB}|\alpha_{ij}\neq 0\}.$$

Thus, each quantum state induces a constraint set

(165) $$C_{\psi }:=\mathrm{Supp}\left(\psi \right)\subseteq U_{AB}.$$

This mapping forgets amplitudes and phases and retains only the structural pattern of correlations between local outcomes.

#### 3.1.3. Separable States and Rectangular Supports

If |ψ〉 is separable, i.e.,

(166) |ψ〉=|ϕ〉⊗|χ〉,

with

(167) $$|\phi \rangle =\sum_{i}u_{i}|r_{i}\rangle ,\;|\chi \rangle =\sum_{j}v_{j}|s_{j}\rangle ,$$

then $\alpha_{ij}=u_{i}v_{j}$ and

(168) $$\mathrm{Supp}\left(\psi \right)=\{\left(r_{i},s_{j}\right)|u_{i}\neq 0,\;v_{j}\neq 0\}=A\times B$$

for some $A\subseteq U_{A}$ and $B\subseteq U_{B}$. Hence,

(169) $$|\psi \rangle \;\mathrm{separable}\;\;⟹\;C_{\psi }\;\mathrm{is}\;a\;\mathrm{Cartesian}\;\mathrm{rectangle}.$$

The converse does not hold in general, reflecting the loss of phase information.

#### 3.1.4. Structural Entanglement via Interaction-Induced Fixed Points

In our framework, composite systems are governed by an approximation operator

(170) $$T_{AB}:=R_{AB*}\circ {K_{AB}}^{*},$$

with fixed-point lattice

(171) $$L_{AB}:=\mathrm{Fix}\left(T_{AB}\right).$$

Local fixed points are embedded by

(172) $$\iota_{A}\left(X\right):=T_{AB}\left(X\times U_{B}\right),\;\iota_{B}\left(Y\right):=T_{AB}\left(U_{A}\times Y\right),$$

and we define

(173) $$S_{AB}:=\langle \iota_{A}\left(L_{A}\right)\cup \iota_{B}\left(L_{B}\right)\rangle .$$

Given a quantum state |ψ〉, we associate the stabilized structure

(174) $$z_{\psi }:=T_{AB}\left(C_{\psi }\right)\in L_{AB}.$$

**Definition** **6.** *A quantum state |ψ〉 is said to be structurally entangled if*

(175) $$z_{\psi }\notin S_{AB}.$$

Thus, structural entanglement arises when the correlation pattern induced by |ψ〉, after stabilization by interaction-dependent approximation, cannot be generated from local fixed points alone.

#### 3.1.5. Example: Bell-Type Correlations

For the Bell state

(176) $$|\Phi^{+}\rangle =\frac{1}{\sqrt{2}}\left(|00\rangle +|11\rangle \right),$$

we obtain

(177) $$C_{\Phi^{+}}=\mathrm{Supp}\left(\Phi^{+}\right)=\left\{\left(0,0\right),\left(1,1\right)\right\},$$

a diagonal constraint set. This constraint coincides with the minimal non-rectangular pattern used in our 2×2 examples and leads, under interaction-dependent interior constraints, to a fixed point that is not generable from local embeddings.

Standard quantum entanglement is, thus, related to our notion of structural entanglement through the mapping

(178) $$\left[\psi \right]⟼C_{\psi }⟼z_{\psi }=T_{AB}\left(C_{\psi }\right),$$

which extracts and stabilizes the correlation structure of quantum states prior to any linear or probabilistic interpretation [37].

Standard quantum entanglement can be understood as a non-factorizable correlation pattern between local measurement outcomes. In our framework, such correlation patterns are represented at the level of row–column relations prior to any linear or probabilistic structure. In particular, the row-set tensor product provides a combinatorial representation of the tensor-product basis of a Hilbert space. Entangled quantum states correspond to constraint sets on these row–column relations that cannot be generated from local row or column sets alone. Our definition therefore captures the structural core underlying quantum entanglement, abstracted from amplitudes and inner products.

### 3.2. Hardy-Type Constraints in Non-Distributive Contexts

A Hardy-type constraint [38,39] represents a specific form of non-classical correlation that demonstrates the impossibility of a local hidden variable explanation through the logic of forbidden outcomes. In our framework, this is represented as an interaction relation $R_{AB}$ that creates a global fixed point which cannot be decomposed into local properties.

#### 3.2.1. Logical Structure of the Constraint

Consider two systems, *A* and *B*, each governed by the $G_{12}$ Peterson–Greechie logic. Let $Context_{A1},Context_{A2}$ be overlapping blocks in System *A*, and $Context_{B1},Context_{B2}$ be blocks in System *B*. We define a Hardy-type constraint by a set of conditional forbiddances for atoms $a_{i}\in L_{A}$ and $b_{j}\in L_{B}$:

1. Contextual Correlation: If outcome $a_{1}$ occurs in $Context_{A1}$, then outcome $b_{1}$ must occur in $Context_{B1}$. This forbids the pair $\left(a_{1},b_{1}^{⊥}\right)$.
2. Cross-Context Requirement: If outcome $b_{1}$ occurs in $Context_{B1}$, then in a different context $Context_{A2}$, the outcome $a_{2}$ must occur. This forbids the pair $\left(b_{1},a_{2}^{⊥}\right)$.
3. The Hardy Paradox: We impose the global interaction constraint that while $a_{1}$ and $b_{2}$ can occur together, the pair $\left(a_{2},b_{2}\right)$ is strictly forbidden in their respective contexts.

#### 3.2.2. Formal Definition via Approximation Operators

In the structural entanglement framework, these rules are encoded into the interaction-induced relation $R_{AB}$ on the product universe $U_{AB}=U_{A}\times U_{B}$.

- The Constraint Set *C*: Let $C\subset U_{AB}$ be the subset of all pairs (a,b) that do not violate the forbiddance rules.
- Stabilization: We apply the interaction-dependent approximation operator $T_{AB}$. A Hardy-type constraint is formally defined as a state where the stabilized fixed point $z_{Hardy}$ satisfies the following:
  (179) $$z_{Hardy}=T_{AB}\left(C\right)=R_{AB*}\left({K_{AB}}^{*}\left(C\right)\right)$$
- Structural Non-Separability: Because $G_{12}$ is non-distributive, the interaction across overlapping blocks (contexts) ensures that
  (180) $$z_{Hardy}\notin \langle \iota_{A}\left(L_{A}\right)\cup \iota_{B}\left(L_{B}\right)\rangle$$

This demonstrates that structural entanglement captures the essence of Hardy’s non-locality theorem. The non-separability of $z_{Hardy}$ arises because the “logical leak” between overlapping blocks in $G_{12}$ prevents the global constraint from being resolved into a join of local propositions $⋁(\iota_{A}\left(x_{i}\right)\wedge \iota_{B}\left(y_{j}\right))$. We obtain a state that is structurally entangled based purely on the algebraic constraints of the orthomodular lattice, without requiring wavefunctions or probability measures.

Here, the term “logical leak” refers to the propagation of contextual constraints across overlapping blocks of the Greechie lattice. A proposition that is locally admissible in one context restricts the admissible propositions in another context through the shared atoms. As a consequence, the stabilization process generated by $T_{AB}$ produces global fixed points that cannot be reconstructed from local contextual structures alone.

**Figure 3:**
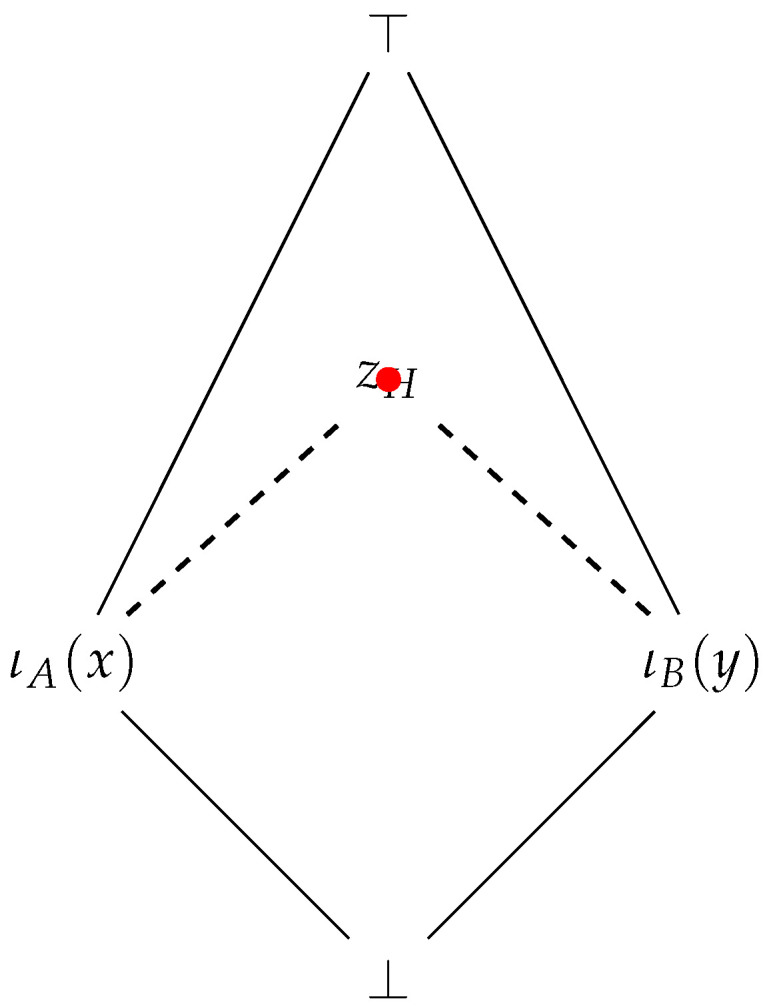
Hasse diagram of the fixed-point lattice $L_{AB}$ for the Hardy-type example. The element $z_{H}$ (highlighted) lies outside the sublattice generated by local embeddings, indicating structural entanglement. The actual sequences show existing order relationships, but the wavy lines indicate the omission of more complex order relationships.

The structure of the fixed-point lattice obtained in this example is visualized in Figure 3. The diagram highlights the position of the entangled fixed point relative to the sublattice generated by local embeddings.

### 3.3. Structural and Relational Entanglement as a New Axis for Quantum Information Theory

Although the present work is primarily theoretical, the proposed characterization of entanglement admits a natural operational interpretation that can, in principle, be accessed experimentally. Instead of relying on full quantum state tomography or a fixed tensor-product decomposition of the Hilbert space, our approach focuses on the structure of observable-dependent correlations encoded in families of measurement contexts.

Concretely, an experimentalist may proceed as follows. Consider a finite set of local or quasi-local observables $\left\{O_{i}\right\}$ accessible in the laboratory. From repeated measurements, one reconstructs the empirical joint statistics and the associated correlation relations among these observables. These correlations define an induced relational structure (or lattice) whose properties can be directly tested without assuming separability at the level of density operators.

The presence of entanglement, in our sense, is witnessed by the failure of this relational structure to admit a classical decomposition compatible with all measurement contexts simultaneously. This criterion provides an experimentally accessible witness that is robust against changes in measurement basis and does not presuppose a particular subsystem partition. As a result, the proposed framework is particularly suited to scenarios involving mixed states, contextual measurements, or complex many-body systems, where standard entanglement witnesses are difficult to implement.

**Proposition** **8** (Relation to separability)**.** *If a bipartite quantum state is separable in the standard sense, then it does not exhibit entanglement according to the proposed relational criterion.*

**Proof.**  For separable states, all joint correlations can be expressed as convex combinations of product correlations. This induces a relational structure that admits a consistent classical decomposition across all measurement contexts, which satisfies the defining condition of non-entanglement in our framework. □

**Proposition** **9** (Relational entanglement beyond PPT)**.** *The proposed relational characterization of entanglement is inequivalent to the positive partial transpose (PPT) criterion. In particular, there exist bipartite quantum states that satisfy the PPT condition and have vanishing standard entanglement monotones, yet exhibit entanglement in the relational sense defined in this work.*

**Proof.**  We demonstrate this inequivalence by constructing an explicit class of states together with a family of measurement contexts for which relational incompatibility arises.

Consider the two-qubit Werner state

(181) $$\rho_{W}\left(p\right)=p|\Psi^{-}\rangle \langle \Psi^{-}|+\left(1-p\right)\frac{I}{4},\;0<p\leq \frac{1}{3},$$

where $|\Psi^{-}\rangle =\left(|01\rangle -|10\rangle \right)/\sqrt{2}$. For this parameter range, $\rho_{W}\left(p\right)$ satisfies the PPT condition and has vanishing negativity, and is therefore not detected as entangled by standard PPT-based criteria.

Now, consider the family of measurement contexts

(182) $$C=\left\{\sigma_{x}⊗\sigma_{x},\;\sigma_{y}⊗\sigma_{y},\;\sigma_{z}⊗\sigma_{z}\right\}.$$

For each context $\sigma_{i}⊗\sigma_{i}\in C$, the correlation function is

(183) $${\langle \sigma_{i}⊗\sigma_{i}\rangle }_{\rho_{W}}=-p,\;i\in \left\{x,y,z\right\}.$$

Hence, for each measurement context individually, the observed correlations admit a classical probabilistic model.

However, we now ask whether there exists a single classical relational structure that can simultaneously reproduce the correlations for all contexts in C. Such a structure would require a joint assignment of classical random variables $\left\{X_{x},X_{y},X_{z},Y_{x},Y_{y},Y_{z}\right\}$ consistent with all observed correlations. Due to the mutual incompatibility of the Pauli observables, $\left[\sigma_{x},\sigma_{y}\right]\neq 0,\;\left[\sigma_{y},\sigma_{z}\right]\neq 0,\;\left[\sigma_{z},\sigma_{x}\right]\neq 0,$ no such joint classical relational model exists.

Therefore, although $\rho_{W}\left(p\right)$ is PPT and undetected by standard entanglement monotones, the family of correlations induced by C cannot be jointly classicalized. This contextual incompatibility witnesses entanglement in the proposed relational sense. □

**Remark** **4.** *Proposition 9 shows that the proposed framework detects a structural and contextual form of entanglement that is invisible to density-operator-based criteria. Unlike PPT or negativity, which depend on a fixed tensor-product decomposition, the present characterization captures the global inconsistency of relational structures across incompatible measurement contexts. This highlights a complementary information-theoretic aspect of entanglement, particularly relevant for mixed states and contextual measurement scenarios.*

**Proposition** **10** (Independence from LOCC monotonicity)**.** *The proposed relational characterization of entanglement is not an entanglement monotone under local operations and classical communication (LOCC). Nevertheless, it provides a well-defined criterion for detecting contextual and structural non-classical correlations.*

**Proof.**  Standard entanglement monotones are defined by their monotonicity under LOCC transformations, reflecting the fact that entanglement cannot be generated by local operations and classical communication alone. Such monotones quantify the amount of entanglement relative to a fixed tensor-product structure of the Hilbert space.

In contrast, the present framework characterizes entanglement in terms of the global consistency of relational structures induced by families of measurement contexts. LOCC operations may alter the accessible set of observables or modify the correlation relations among them, thereby changing the associated relational structure. As a result, relational entanglement need not be monotonic under LOCC transformations.

This non-monotonicity does not indicate an inconsistency of the framework. Rather, it reflects the fact that the proposed criterion probes a different aspect of quantum correlations, namely, the impossibility of jointly classicalizing correlations across incompatible measurement contexts, which is not captured by LOCC-based quantification schemes. □

**Remark** **5.** *Propositions 9 and 10 clarify that the present framework does not aim to replace standard entanglement measures. Instead, it introduces a complementary notion of entanglement that is sensitive to contextual and structural features of quantum correlations, rather than to resource convertibility under LOCC.*

#### Scope of the Proposed Notion of Entanglement

At this point, it is important to clarify the scope of the proposed notion of entanglement. The present framework is not intended to refine or compete with standard resource-theoretic criteria such as PPT or LOCC-based entanglement monotones. Those criteria are designed to quantify entanglement with respect to a fixed tensor-product structure and operational convertibility under local operations and classical communication. In contrast, our definition targets a complementary aspect of non-classicality: the impossibility of jointly classicalizing relational structures induced by families of incompatible measurement contexts. Accordingly, a quantum state may satisfy the PPT condition or exhibit vanishing LOCC-based entanglement measures, while still being entangled in the structural and relational sense defined here. This distinction is essential for understanding the results of Propositions 7 and 8, and motivates the subsequent interpretation in terms of relational databases and fixed-point semantics.

Unlike the sheaf-theoretic framework of Abramsky and Brandenburger [10], which focuses on the existence or non-existence of global sections, the present framework studies the entire lattice of stabilized global relations. Structural entanglement is therefore characterized not merely by the absence of a global model, but by the existence of fixed points that cannot be generated from local embeddings.

### 3.4. Structural Entanglement and Relational Databases

Relational databases offer a precise and operational framework for describing how local pieces of information combine into global structures under constraints. This perspective has been fruitfully exploited in the foundations of quantum theory, most notably in the relational and sheaf-theoretic approach of Abramsky and Brandenburger [10], where non-locality and contextuality are understood as obstructions to the existence of a globally consistent relational model assembled from local data.

The present framework can be seen as a lattice-theoretic generalization of this idea. Rather than asking only whether a global relational structure exists, we study the entire space of stabilized global relations generated by interaction-dependent approximation operators. Within this space, entanglement is identified as a structural feature: the impossibility of generating certain global fixed points from local components alone.

#### 3.4.1. Relational Databases as Composite Information Systems

A relational database instance may be viewed as a finite set of tuples over a Cartesian product of attribute domains. In our notation:

- Local systems correspond to attribute domains $U_{A}$ and $U_{B}$.
- The composite universe is the Cartesian product $U_{AB}=U_{A}\times U_{B}$.
- A database relation is a subset $C\subseteq U_{AB}$, representing admissible combinations of attribute values.

From this viewpoint, a relation already functions as a constraint: it specifies which global combinations are allowed. This interpretation aligns directly with the role played by constraint sets in the construction of composite systems in the present framework.

As mentioned before, the set of all fixed points, Fix(T), forms a complete lattice under set inclusion. In database terms [40], these fixed points represent stable relational instances that are fully consistent with the imposed interaction rules.

In relational database language, our structural entanglement means the following:

A relation is structurally entangled if it cannot be generated from stabilized local relations using projections, joins, and lattice combinations.

This notion closely parallels Abramsky’s characterization of contextuality as the impossibility of gluing local sections into a global one. However, instead of a binary distinction between consistency and inconsistency, the present framework yields a graded structure: entangled relations occupy specific positions in the fixed-point lattice beyond the sublattice of locally generable relations.

#### 3.4.2. Relation to Abramsky’s Relational Approach

In Abramsky’s framework, a measurement scenario is modeled as a database schema, contexts correspond to tables, and compatibility on overlaps is expressed via joins. Contextuality arises when no global relation exists whose projections reproduce all local tables.

The present approach agrees at the relational level but differs in emphasis:

1. Existential questions about global sections are replaced by a fixed-point semantics classifying all stabilized global relations.
2. No probabilistic or presheaf structure is presupposed; relations and closure operators suffice.
3. Entanglement is identified with non-generability from local embeddings, rather than merely with inconsistency.

In this sense, Abramsky’s contextuality appears as a special case in which stabilized relations necessarily lie outside the locally generated sublattice.

#### 3.4.3. Implications for Database Theory and Information Processing

From the viewpoint of database theory, structural entanglement corresponds to irreducible join patterns: relations that cannot be decomposed into joins of their projections without loss of information. The lattice of fixed points provides a natural semantic framework for understanding such irreducibility under constraints and indiscernibility.

More broadly, this interpretation suggests that entanglement is not an exclusively quantum phenomenon, but a general structural feature of composite information systems. Relational databases therefore provide a concrete domain in which entanglement-like phenomena can be studied independently of Hilbert spaces or quantum states.

Our framework of structural entanglement integrates naturally with the relational database perspective pioneered by Abramsky and collaborators. By focusing on interaction-induced stabilization and non-generability from local components, it extends the relational view of contextuality into a richer structural theory. This opens the door to applications not only in quantum foundations, but also in database theory, logic, and generalized information processing.

#### The Example: A 2×2 Relational Database

Consider two attributes (local systems) *A* and *B* with domains $U_{A}=\left\{a_{1},a_{2}\right\}$ and $U_{B}=\left\{b_{1},b_{2}\right\}$, as shown in Table 1. We define a global relation $D_{0}\subseteq U_{A}\times U_{B}$ that represents a perfect correlation, analogous to a Bell state.

In the Abramsky and Brandenburger framework, the focus is on global consistency and sheaf sections.

- Local Projections: The projections are $\pi_{A}\left(D_{0}\right)=\left\{a_{1},a_{2}\right\}$ and $\pi_{B}\left(D_{0}\right)=\left\{b_{1},b_{2}\right\}$.
- Contextuality: Contextuality is defined as an *obstruction* to extending local sections to a global section.
- Result: For this single relation $D_{0}$, it is considered a “global section”. In Abramsky’s view, one typically needs a set of relations across different measurement contexts (like a Bell-CHSH scenario) to demonstrate that no single global table can satisfy all marginals.

In our approach, the focus is on non-generability from local embeddings within a lattice of fixed points.

- Stabilization: We apply a closure operator $T_{AB}$ that accounts for interaction constraints and indiscernibility. This defines a lattice of “fixed points” (stabilized relations).
- Local Embeddings: Let $\iota_{A}$ and $\iota_{B}$ be the embeddings of local information into the global system. The sublattice $S_{AB}$ is generated by all possible combinations of these local embeddings:
  $$S_{AB}=\langle \iota_{A}\left(U_{A}\right),\iota_{B}\left(U_{B}\right)\rangle .$$
- Entanglement: If $D_{0}$ is a fixed point of the global system but cannot be expressed as a join/meet of elements in $S_{AB}$, it is structurally entangled.
- Result: Even if the local logic is Boolean, $D_{0}$ is entangled if the “interaction” creates a fixed point that is not reachable by simply stacking local data.

The relationship between Abramsky’s approach and our structural entanglement is summarized in Table 2.

## 4. Concluding Discussion

The proposed framework should not be viewed as a replacement for existing approaches to generalized entanglement, contextuality, or generalized probabilistic theories. Rather, it introduces a complementary perspective in which interaction-induced fixed points play the role of fundamental informational objects. From this viewpoint, non-separability emerges as a property of lattice-theoretic stabilization rather than of state vectors, probability distributions, or categorical morphisms.

This work establishes a lattice-theoretic foundation for composite systems that defines entanglement as a structural property arising from interaction-dependent closure and interior operators. By moving away from the prerequisite of Hilbert spaces, we demonstrate that non-separability is a general feature of information structures where global fixed points cannot be reconstructed from local embeddings. We emphasize the following key findings of our paper: Structural independence: Entanglement does not inherently require non-distributive or orthomodular logic. Our results show that entangled fixed points can emerge even when local lattices are strictly Boolean.

Emergence via interaction: While simple interaction-induced closures are insufficient to produce entanglement in minimal 2×2 systems, the introduction of interaction-dependent interior constraints (lower approximations) triggers the emergence of entangled fixed points. A unified perspective: The framework successfully bridges abstract lattice theory and standard quantum mechanics. By treating quantum states as correlation patterns (supports) within a row-set tensor product, we identify the “structural core” of quantum entanglement independent of complex amplitudes or probabilities. We also point to the following foundational implications: The mapping $\left[\psi \right]\mapsto C_{\psi }\mapsto z_{\psi }$ provides a method to stabilize the correlation structure of quantum states. We have shown that the Bell state $\Phi^{+}$ induces a diagonal constraint set that leads to a fixed point non-generable from local components, formally aligning our structural definition with physical reality. This suggests that the essence of quantum non-locality is a manifestation of how interactions constrain the discernibility of global states. At the same time we should point to the following limitations (stimulating future research):

While the current model effectively captures the structural patterns of pure states and their stabilized supports, it intentionally “forgets” phase and amplitude information. Consequently, while every separable quantum state corresponds to a separable structural element, the converse does not always. Future research will focus on the following: Refining the mapping: Investigating if additional lattice constraints can recover phase-dependent properties. Computational complexity: Analyzing the generation of fixed-point lattices in higher-dimensional N×N systems. Generalized contextuality: Applying this interaction-induced fixed-point logic to model context-dependent information processing in non-physical domains, such as cognitive science or abstract computation. In conclusion, our framework offers a robust generalization of entanglement that identifies interaction-induced fixed points as the fundamental unit of non-separability, providing a bridge between combinatorial logic and quantum information theory.

## Figures and Tables

**Table 1:** The global relation $D_{0}$ (diagonal correlation).

Tuple ID	Attribute *A*	Attribute *B*
1	$a_{1}$	$b_{1}$
2	$a_{2}$	$b_{2}$

**Table 2:** Comparison of Abramsky’s approach and structural entanglement.

Feature	Abramsky (Sheaf)	Structural Entanglement
**Ontology**	Bundle of local sections	Lattice of fixed points
**Mechanism**	Compatibility of projections	Generability from embeddings
**Entanglement**	Global inconsistency	Non-generability
**Key Concept**	Obstruction/sheaf cohomology	Interaction-induced fixed points

## Data Availability

The original contributions presented in this study are included in the article. Further inquiries can be directed to the corresponding author.

## Appendix A. The Peterson–Greechie Quantum Logic

In the field of quantum foundations, “quantum logic” refers to a lattice of propositions that is orthomodular and, in general, non-distributive. While the standard quantum logic is given by the lattice of closed subspaces of a Hilbert space, finite orthomodular lattices provide important examples for testing which features of quantum logic depend on Hilbert-space representability. The Greechie lattice $G_{12}$ is one such finite example.

### Definition: The Greechie Lattice G_*12*_ [19,41,42]

The lattice $G_{12}$ considered here is the twelve-element orthomodular lattice introduced by Greechie. It can be represented as the pasting of two Boolean algebras, each isomorphic to $2^{3}$, along a common Boolean subalgebra isomorphic to $2^{2}$. Equivalently,

(A1) $$|G_{12}|=8+8-4=12.$$

Let

(A2) $$U=\{a_{1},a_{2},a_{3},a_{4},a_{5}\}.$$

The elements of $G_{12}$ may be represented as the following subsets of *U*:

(A3) $$\begin{array}{cc} G_{12}= & \{⌀,\left\{a_{1}\right\},\left\{a_{2}\right\},\left\{a_{3}\right\},\left\{a_{4}\right\},\left\{a_{5}\right\},\\ & \;\;\left\{a_{1},a_{3}\right\},\left\{a_{2},a_{3}\right\},\left\{a_{3},a_{4}\right\},\left\{a_{3},a_{5}\right\},\\ & \;\;\;\{a_{1},a_{2},a_{4},a_{5}\},U\}. \end{array}$$

The two Boolean blocks are

(A4) $$B_{1}=\left\{⌀,\left\{a_{1}\right\},\left\{a_{2}\right\},\left\{a_{3}\right\},\left\{a_{1},a_{2}\right\},\left\{a_{1},a_{3}\right\},\left\{a_{2},a_{3}\right\},\left\{a_{1},a_{2},a_{3}\right\}\right\},$$

and

(A5) $$B_{2}=\left\{⌀,\left\{a_{3}\right\},\left\{a_{4}\right\},\left\{a_{5}\right\},\left\{a_{3},a_{4}\right\},\left\{a_{3},a_{5}\right\},\left\{a_{4},a_{5}\right\},\left\{a_{3},a_{4},a_{5}\right\}\right\}.$$

In the pasted lattice, the two Boolean algebras share the four-element Boolean subalgebra

(A6) $$B_{1}\cap B_{2}=\left\{⌀,\left\{a_{3}\right\},\left\{a_{1},a_{2},a_{4},a_{5}\right\},U\right\}.$$

Thus, $G_{12}$ is obtained by identifying the common bottom element, the common top element, the shared atom $\left\{a_{3}\right\}$, and its orthocomplement $\left\{a_{1},a_{2},a_{4},a_{5}\right\}$.

The orthocomplementation is given by

(A7) $${⌀}^{⊥}=U,$$

(A8) $${\left\{a_{3}\right\}}^{⊥}=\left\{a_{1},a_{2},a_{4},a_{5}\right\},$$

(A9) $${\left\{a_{1}\right\}}^{⊥}=\left\{a_{2},a_{3}\right\},$$

(A10) $${\left\{a_{2}\right\}}^{⊥}=\left\{a_{1},a_{3}\right\},$$

(A11) $${\left\{a_{4}\right\}}^{⊥}=\left\{a_{3},a_{5}\right\},$$

and

(A12) $${\left\{a_{5}\right\}}^{⊥}=\left\{a_{3},a_{4}\right\}.$$

The remaining complements are determined by involution:

(A13) $${\left(x^{⊥}\right)}^{⊥}=x.$$

This lattice is orthomodular but non-distributive. The non-distributivity arises because the two Boolean blocks are pasted along a common four-element Boolean subalgebra rather than forming a single Boolean algebra. For example, elements chosen from different Boolean blocks need not satisfy the distributive law

(A14) x∧(y∨z)=(x∧y)∨(x∧z).

Hence, $G_{12}$ is a minimal finite example of a non-Boolean quantum logic obtained by structural pasting of Boolean contexts.

In the context of structural entanglement, $G_{12}$ provides the local logic $L_{A}$ and $L_{B}$. Entanglement is demonstrated when the composite fixed-point lattice $L_{AB}$ contains global propositions that cannot be resolved into the intersecting contexts of the local Peterson–Greechie structures.

The Peterson–Greechie lattice $G_{12}$ is classified as a quantum logic because it satisfies the algebraic requirements of orthomodularity while exhibiting the non-classical feature of non-distributivity.

## Appendix B. Breakdown of Transitivity as the Essence of Hardy’s Paradox

To understand why considered above classical chain of logic breaks down, we have to look at the difference between context-independent truth and context-dependent properties. In classical (Boolean) logic, we assume a context-independent reality where properties possess fixed truth values (1 or 0) regardless of the measurement context.

- The Classical View: In a distributive lattice, if $a_{1}\Rightarrow b_{1}$ and $b_{1}\Rightarrow a_{2}$, the law of transitivity dictates that $a_{1}\Rightarrow a_{2}$. If we observe $a_{1}$ and $b_{2}$ occurring together, and we know $a_{1}$ forces $a_{2}$, we must conclude that $a_{2}$ and $b_{2}$ are compatible and can occur together.
- The Quantum-Logical Break: In the Peterson–Greechie logic $G_{12}$, the properties $a_{1}$ and $a_{2}$ belong to different, incompatible contexts (maximal Boolean blocks). The “paradox” arises because
  1. The first implication ($a_{1}\Rightarrow b_{1}$) is established within $Context_{1}$.
  2. The second implication ($b_{1}\Rightarrow a_{2}$) is established within $Context_{2}$.

Because $a_{1}$ and $a_{2}$ are incompatible, they cannot be assigned simultaneous truth values in a way that respects the distributive law. The interaction $R_{AB}$ forces a global correlation that respects the “loops” in the $G_{12}$ structure.

The paradox is the realization that the global fixed point $z_{Hardy}$ contains the pair $\left(a_{1},b_{2}\right)$ but strictly excludes $\left(a_{2},b_{2}\right)$. This configuration is mathematically impossible if the system were a simple join of local Boolean lattices. This confirms that the “leak” or overlap between contexts in the Greechie lattice is the algebraic source of structural entanglement. It proves that the global state $z_{Hardy}$ cannot be explained by local, context-independent properties assigned to System *A* and System *B* individually.

### Appendix B.1. The Classical (Boolean) View

In classical logic, we assume that every property has a fixed value (1 or 0) at all times, regardless of how we measure it. If $a_{1}\Rightarrow b_{1}$, it means that every time $a_{1}$ is true, $b_{1}$ is true. If $b_{1}\Rightarrow a_{2}$, it means that every time $b_{1}$ is true, $a_{2}$ is true. Therefore, by transitivity, $a_{1}\Rightarrow a_{2}$. If we then find a case where $a_{1}$ and $b_{2}$ are both true, and we already know that $a_{1}$ forces $a_{2}$, then $a_{2}$ and $b_{2}$ must be true together.

### Appendix B.2. The Quantum (Non-Distributive) Break

In the $G_{12}$ logic (and quantum mechanics), the “properties” $a_{1}$ and $a_{2}$ are incompatible. They belong to different “contexts” (different blocks in the Greechie lattice). The “paradox” happens because of the following:

- The First Link ($a_{1}\Rightarrow b_{1}$): This is a correlation established in one context (let’s call it Context 1).
- The Second Link ($b_{1}\Rightarrow a_{2}$): This correlation is established in a different context (Context 2).
- The Break: Because $a_{1}$ and $a_{2}$ are incompatible, they cannot be assigned “truth values” at the same time in a way that respects the distributive law.

In a distributive (Boolean) lattice, we can always draw a single “map” (a Venn diagram) of all possibilities. In a Peterson–Greechie lattice, we have multiple “maps” that overlap at specific points, but we cannot flatten them into one single map.

### Appendix B.3. The Essence of the Hardy Constraint

The “Hardy-type” constraint exploits this. It sets up a situation where

- If we look at the system through the “lens” of Context 1, $a_{1}$ forces $b_{1}$.
- If we switch your “lens” to Context 2, $b_{1}$ forces $a_{2}$.
- But the global constraint $R_{AB}$ (the Interaction) forbids $a_{2}$ and $b_{2}$ from ever happening together.

The “Paradox” Explained:
If the world were classical, one would say: “Wait, if I see $a_{1}$ and $b_{2}$, then $a_{2}$ MUST be there, but you just told me $a_{2}$ and $b_{2}$ are forbidden! That’s a contradiction!”

In the Peterson–Greechie logic, it is not a contradiction; it is a structural entanglement. It proves that the state of the system cannot be explained by “local properties” assigned to A and B independently. The “leak” between the overlapping blocks in the Greechie lattice allows $a_{1}$ and $b_{2}$ to coexist without forcing $a_{2}$ to be “true” in a way that classical logic would require.

This is why $z_{Hardy}$ is not in the sublattice $S_{AB}$.

- $S_{AB}$ is what we get if we try to build the world out of local, independent “blocks”.
- $z_{Hardy}$ is a fixed point that exists only because the interaction $R_{AB}$ spans across the “seams” where the different local contexts of $G_{12}$ meet.
- It is “entangled” because the global truth (the fixed point) is strictly larger and more complex than the sum of the local truths.

(A15)
$$z_{Hardy}\in L_{AB},\;z_{Hardy}\notin \langle \iota_{A}\left(L_{A}\right)\cup \iota_{B}\left(L_{B}\right)\rangle$$

This result demonstrates that structural entanglement captures the essence of non-locality without requiring wavefunctions or probability measures, relying instead on the rigid algebraic constraints of orthomodular lattices.
